# A Rare Clinical Case: Giant Splenic Artery Aneurysm and Its Successful Endovascular Treatment

**DOI:** 10.1155/2017/3537083

**Published:** 2017-07-13

**Authors:** Alptekin Yasim, Hakan Kara, Erdinc Eroglu

**Affiliations:** ^1^Faculty of Medicine, Kahramanmaras Sutcu Imam University, Kahramanmaras, Turkey; ^2^Giresun Ada Hospital, Giresun, Turkey

## Abstract

**Objectives:**

The true giant splenic artery aneurysms are extremely rare and can be fatal. Although surgical approach has been the standard of care in the past, endovascular methods gained more importance in recent years.

**Background:**

We describe a case with true giant splenic artery aneurysm, treated with endovascular approach.

**Methods:**

A 68-year-old female patient with abdominal pain admitted to our clinic had true splenic artery aneurysm (14 × 10 × 9 cm). We decided on endovascular treatment using vascular plug and the treatment was performed successfully.

**Conclusions:**

Due to high mortality and morbidity in open surgery, endovascular treatment of giant splenic artery aneurysm is a better treatment option.

## 1. Introduction

The splenic artery aneurysm (SAA) is the third most common intraabdominal aneurysm following abdominal aorta aneurysms and iliac artery aneurysm. It constitutes more than 60% of the all visceral artery aneurysms [1–4]. It is reported 0.8–1% in prevalence arteriography and 0.001–0.2% in autopsy series [1, 5, 6]. Although most cases are asymptomatic, risk of rupture increases in aneurysms larger than 2 cm. The risk of rupture ranges from 2% to 10% [1, 5–7] and risk increases in presence of portal hypertension, in liver transplant patients, and during pregnancy [1, 5, 8, 9]. In addition, the risk of rupture is high in men and in smokers [7]. Occasionally aneurysm can erode on adjacent organs or into pancreatic duct and present as gastrointestinal bleeding [2]. Rupture is the catastrophic complication of SAAs and causes 10–100% mortality [1, 4, 5, 7].

In order to prevent risk of rupture, ACC/AHA guidelines recommended that treatment is indicated in asymptomatic aneurysm that is larger than 2 cm [7]. Additionally, treatment is indicated in patients with symptomatic aneurysm, pregnancy and portal hypertension and woman in childbearing age even if the aneurysm is smaller than 2 cm [1, 8].

SAAs are generally smaller than 3 cm. If aneurysm is greater than 10 cm, they are called giant SAA. These aneurysms can be true or pseudoaneurysms. But aneurysms greater than 10 cm are rarely seen.

Surgery (open or laparoscopic) has been used as treatment method for many years. But recently endovascular treatments (stent-graft implantation, coil embolization, plug deployment, or glue embolization) are used as these treatment methods are often more successful. The purpose of this study is to present a case with true giant splenic artery aneurysm that had endovascular treatment.

## 2. Case Report

A 68-year-old woman patient was referred to our clinic following detection of abdominal aneurysm after USG examination. She suffered from abdominal discomfort. She was congenitally deaf and dumb. Other physical findings were normal. Her past medical history revealed occasional abdominal pain and constipation for the past 6 months. There was no risk factor such as hypertension, diabetes, smoking, or atherosclerosis. Patient was not using any medical treatment and had no history of trauma or surgery. There was no history of any systemic infections, family history of aneurysm, and connective tissue disorders. She was nullipara.

All laboratory tests were within the normal range.

**Figure 1:**
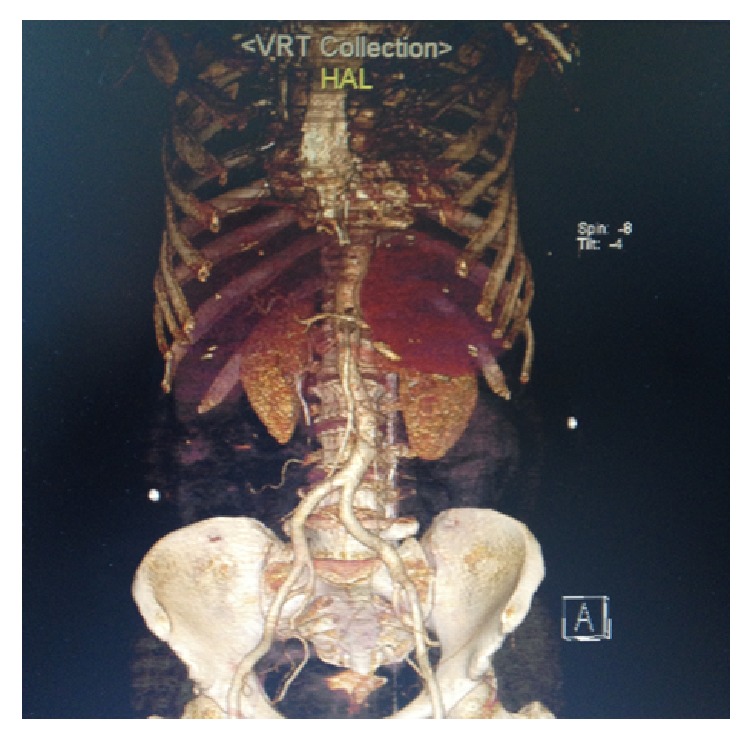
Computed tomographic angiography revealed aneurysmatic expansion.

A computed tomographic angiography revealed 14 × 10 × 9 cm sized regular bounded aneurysmatic expansion placed between pancreas tail, spleen, stomach, and kidney in left upper quadrant of abdomen. It causes compression on pancreas tale, probably splenic artery sourced and in close connection with distal part of splenic artery bifurcation (Figure 1).

**Figure 2:**
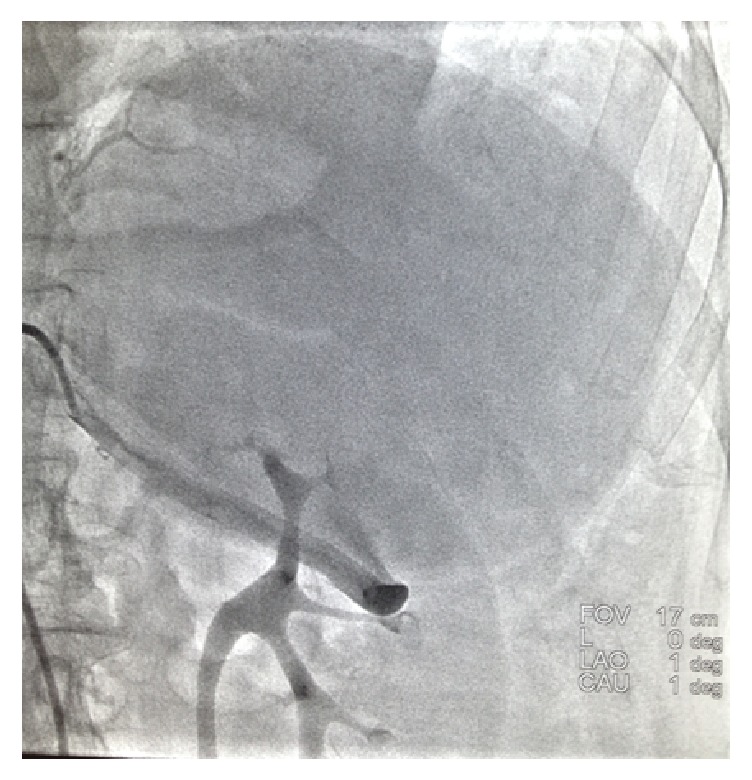
Angiogram showed giant splenic artery aneurysm.

A selective angiogram was performed and showed splenic artery expanding to 6.5 mm diameter and confirmed the presence of true giant saccular splenic aneurysm arising from splenic artery and expanding to the superior (Figure 2), after aneurysm splenic artery became rudimentary.

**Figure 3:**
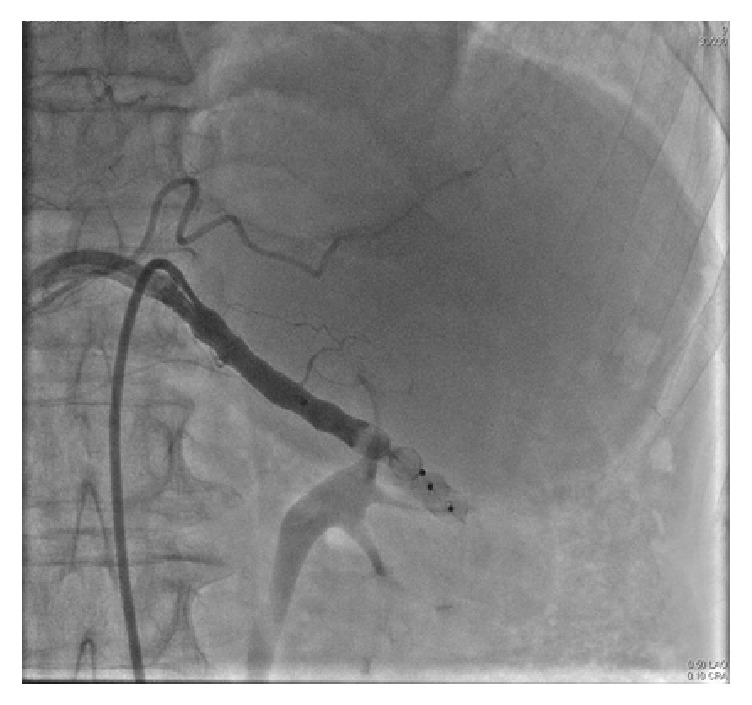
Angiogram after vascular plug employment.

The patient was scheduled for endovascular intervention. Stent-graft implantation was not possible due to artery diameter being too wide at proximal portion and small in distal portion. Coil or glue embolization also was not possible due to wide aneurysm. Eventually, to close the feeding artery with vascular plug is decided. A right common femoral artery approach was performed under local anesthesia; a 7-French sheath was placed. A 7-French guiding catheter was positioned at the origin of the splenic artery over 0.035′′  × 260 cm guidewire. Splenic angiogram was obtained again and reference values were recorded by contrast injection. After 7 mm balloon expanded inside of the splenic artery over 0.014′′ inch floppy wire and long injection performed to celiac trunk. No retrograde flow was observed inside of the aneurysm. 8 mm Amplatzer vascular plug (St. Jude Medical, Austin, TX) was deployed in aneurysm entry. Minimal leakage was observed after contrast injection. A 10 mm Amplatzer vascular plug was deployed proximally of the 8 mm plug. No retrograde filling in to the aneurysm and no leakage were observed in contrast injection from celiac trunk (Figure 3).

**Figure 4:**
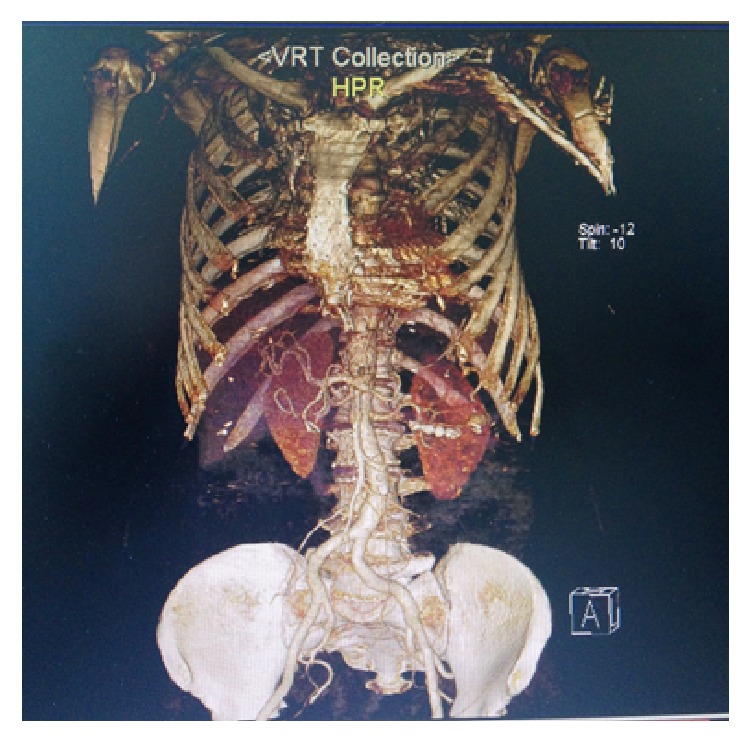
After three months, CT angiography showed thrombosed aneurysm sack and normal spleen.

Patient was discharged at second day after the procedure. At the third month, CT angiography showed thrombosed aneurysm sack and no problem with spleen (Figure 4). Patient was clinically comfort with no complaint.

## 3. Discussion

SAAs are the most common visceral artery aneurysms and their etiology is unclear. Pregnancy, portal hypertension, cirrhosis, liver transplantation, hypertension, atherosclerosis, medial fibrodisplasia, splenomegaly, pancreatic pseudocyst, and vascular collagen diseases are the risk factors for SAA development [1, 2, 4, 8].

Although it is more common in women 4 times greater than men, rupture is more common in men [4]. Regarding the developments and more common usage of medical imaging technologies, we encounter small SAAs more often and treat these small SAAs successfully with endovascular methods. SAAs are generally smaller than 3 cm and they are called giant SAAs if aneurysm is greater than 10 cm [2, 3]. Giant SAAs are extremely rare cases. Only 12 giant SAAs were reported in the literature search in 2005 [3]. However, it is not reported that these aneurysms are true or pseudoaneurysms. Recently, in another study, 10 true giant SAAs were reported [6].

These giant aneurysms were treated by surgery in the past years [2, 3] due to lack of endovascular techniques. It is nearly impossible to treat giant SAAs with stent grafts due to place of occurrence of SAA that is generally located at distal portion of splenic artery, because the diameter difference before and after aneurysm is great and there is no enough place on the distal to aneurysm to implant stent graft unlike in our case.

In this case due to large diameter of aneurysm sack and high flow, coil embolization was not an option. Guziński et al. treated a giant aneurysm with glue embolization into splenic artery after cutting off the blood flow [6]. We planned to cut off arterial flow by vascular plug. The important point here was whether or not spleen will go to autosplenectomy. However, splenectomy was performed in most of the surgical procedures in the past. Although we were going to cut off blood flow in spleen without splenectomy, there was a risk of splenic infarct. However spleen has rich collateral and short gastric artery feeding; it is reported that this may develop splenic infarct and postembolization syndrome [5, 9, 10]. But it is mentioned that after this procedure splenic insufficiency is rare [4]. Li et al. performed aneurysm repair to 35 patients with SAA by preserving splenic artery in some of them, whereas they occluded splenic artery in the rest of the patients in their study [5]. Postembolization syndrome consists of abdominal pain, fever, and vomiting symptoms occurring in 15% of the patients where they preserved splenic artery and 37% of the patients where they did not. However, they reported no splenic artery insufficiency in both groups and it is safe to perform embolization in splenic artery. We occluded splenic artery from the proximal portion of the aneurysm in the light of these findings. We did not observe postembolization syndrome with our patient.

## 4. Conclusion

This case report is unique endovascular treatment by vascular plug of giant SAA in the literature. Splenic artery can be occluded safely in these particular cases. By these ways, patients exposure to surgical morbidity and mortality can reduce.
